# Molecular epidemiology and pretreatment drug resistance of HIV-1 among newly diagnosed individuals in Nanning City, Guangxi, China

**DOI:** 10.1128/spectrum.03149-24

**Published:** 2025-09-12

**Authors:** Ting Huang, Jinfeng He, Qiuqian Su, Liangjia Wei, Jiao Qin, Xinju Huang, Chunxing Tao, Fei Zhang, Li Ye, Ping Cen, Hao Liang, Bingyu Liang

**Affiliations:** 1 Guangxi Key Laboratory of AIDS Prevention and Treatment, School of Public Health, Guangxi Medical University 654463, Nanning, Guangxi, China; 2 Biosafety III Laboratory, Life Sciences Institute, Guangxi Medical University 666892, Nanning, Guangxi, China; 3 The Fourth People’s Hospital of Nanning, Guangxi, China; Barnard College, Columbia University, New York, New York, USA

**Keywords:** HIV, pretreatment drug resistance (PDR), drug resistance mutations (DRMs), molecular transmission networks

## Abstract

Emerging pretreatment drug resistance (PDR) significantly reduces the effectiveness of HIV antiretroviral therapy (ART). This study assessed the prevalence, associated factors, and transmission networks of PDR among newly diagnosed, ART-naïve individuals in Guangxi, China, from 2019 to 2022. A cross-sectional study was conducted between 2019 and 2022 in Nanning, Guangxi, involving 1,260 newly diagnosed, ART-naïve individuals with HIV. PDR levels and mutations were identified using the Stanford HIV Drug Resistance Database. Multivariable logistic regression models were employed to identify factors associated with PDR and to cluster the molecular network. A total of 1,048 eligible pol sequences were analyzed. The overall prevalence of PDR was 8.4%, with non-nucleoside reverse transcriptase inhibitors (NNRTIs) being the most commonly affected drug class. The most prevalent NNRTI-associated mutation was E138A (2.4%). High-level resistance was primarily observed to efavirenz and nevirapine. The CRF08_BC subtype exhibited significant clustering of PDR-related sequences. Those individuals diagnosed in 2022 were more likely to have PDR. Transmission networks clustering was significantly associated with CRF01_AE and CRF08_BC subtypes, older age, and heterosexual transmission. This study identified a moderate prevalence of PDR among newly diagnosed HIV patients in Guangxi, primarily driven by NNRTI-associated resistance mutations. The pronounced clustering of PDR in the CRF08_BC subtype highlights the need for subtype-specific surveillance and intervention strategies. To improve treatment outcomes and constrain the spread of resistance, targeted educational programs on ART adherence and drug resistance awareness should be prioritized, especially among older adults and individuals infected through heterosexual contacts, alongside enhanced molecular monitoring.

**IMPORTANCE:**

This study highlights the growing challenge of pretreatment drug resistance (PDR) among newly diagnosed individuals with HIV in Nanning City. As antiretroviral therapy (ART) coverage expands, the persistence and transmission of drug-resistant strains pose a significant barrier to long-term treatment success. By documenting trends in PDR and identifying associated factors within a large representative sample, this study offers timely and actionable insights for clinicians and public health policymakers. The identification of key resistance mutations and clustering patterns, particularly in the CRF08_BC subtype, provides a critical foundation for tailored intervention strategies. Overall, these findings address a significant regional data gap and contribute to the optimization of HIV treatment and prevention efforts in China.

## INTRODUCTION

Antiretroviral therapy (ART) has been highly effective in suppressing HIV viral loads to undetectable levels, significantly reducing transmission and transforming acquired immunodeficiency syndrome (AIDS) into a manageable chronic disease (1). However, the emergence of drug resistance during ART remains a significant challenge for both HIV treatment and prevention. Pretreatment drug resistance (PDR) refers to resistance detected in individuals who are ART-naïve or have prior exposure to antiretroviral drugs. PDR can compromise the effectiveness of ART, accelerate disease progression, increase mortality, and facilitate secondary HIV transmission. The rising prevalence of resistance, particularly to non-nucleoside reverse transcriptase inhibitors (NNRTIs), has been widely documented (2, 3). Notably, efavirenz (EFV) and nevirapine (NVP) are associated with higher levels of resistance compared to other NNRTIs (4, 5), prompting the World Health Organization (WHO) to recommend prioritizing PDR surveillance in countries that use these drugs as part of their first-line ART regimens (6). Therefore, ongoing surveillance of PDR prevalence, resistance patterns, and transmission dynamics is critical for optimizing HIV preventive strategies and ensuring the long-term effectiveness of ART programs.

In 2003, China launched its National Free Antiretroviral Treatment Program (7). By 2016, the “Treat-all” policy was implemented, extending ART access to all consenting people living with HIV (PLWH). ART implementation in China has significantly reduced all-cause mortality among PLWH, from 5.4% in 2013 to 2.7% in 2022 (8). However, a substantial number of PLWH continue to die from AIDS-related complications, underscoring the importance of investigating contributing factors, including drug resistance, that compromise treatment effectiveness. Previous studies have shown that PDR is increasingly prevalent in several regions of China, with reported rates of 23.1% in Shenzhen City (9), 18.3% in Xi’an City (10), 17.4% in Shanghai City (11), and 10.0% in Hunan Province (12). These findings highlight the necessity for continuous surveillance of PDR trends to inform and enhance HIV prevention and treatment strategies.

Molecular transmission networks provide critical insights into the dissemination pathways of drug-resistant HIV strains within populations. Studies from Croatia (13) and Mexico (14) have shown that resistant strains frequently circulate within tightly connected transmission networks. Similarly, the emergence of drug resistance clusters in certain epidemic regions of China indicates ongoing transmission of resistant HIV strains among PLWH (15, 16).

In Guangxi, molecular network studies on PDR have been conducted in several regions, including Qinzhou and Baise (2). This study revealed that individuals over the age of 50 were more likely to be part of transmission clusters (TCs) and that the CRF08_BC subtype was particularly prone to sharing drug resistance mutations (DRMs) (2). These findings highlight the urgent need to strengthen surveillance and implement targeted interventions. Understanding regional differences in the dynamics of drug resistance is essential for optimizing treatment strategies and guiding the development of new antiretroviral drugs. However, existing studies have not comprehensively covered all areas, leaving significant gaps that may distort the overall picture of drug resistance in the province.

Therefore, this study aims to investigate the prevalence of primary PDR and to explore the transmission factors of DRM within molecular networks by recruiting newly diagnosed, ART-naive individuals, thereby providing essential evidence to inform and enhance HIV control strategies.

## RESULTS

### Sociodemographic and clinical characteristics of the participants

**Fig 1:**
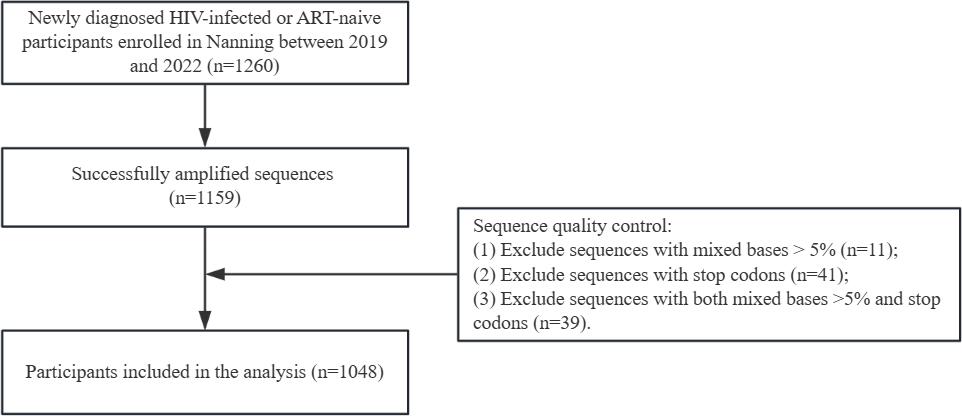
Patient enrollment flowchart. ART, antiretroviral therapy.

In this study, we enrolled 1,260 newly diagnosed HIV-infected individuals. Among them, 1,159 pol gene sequences were successfully amplified. Of these, 11 sequences contained mixed bases >5%, 41 contained stop codons, and 39 contained both mixed bases >5% and stop codons. After applying quality control criteria, 1,048 sequences were included in the final analysis (Fig. 1). As shown in Table 1, the majority of participants were aged 50–69 years. Most were male (73.3%) and of Zhuang ethnicity (65.9%). In terms of education, 52.8% had completed only primary school or below. Regarding marital status, 51.2% of participants were married. Most participants were farmers (76.5%). Heterosexual transmission was the predominant route of HIV transmission (92.5%). Additionally, 57.4% had CD4^+^ T-cell counts of ≥200 cells/mm^3^ before initiating ART, and 39.2% were infected with the CRF01_AE subtype.

**TABLE 1:** Sociodemographic and clinical characteristics of HIV-positive individuals^*a*^

Characteristic	Total (*N* = 1048)	CRF01_AE (*N* = 411)	CRF07_BC (*N* = 287)	CRF08_BC (*N* = 301)	Other subtypes (*N* = 49)
Age (years)
18–34	126 (12.0%)	27 (6.6%)	65 (22.6%)	14 (4.7%)	20 (40.8%)
35–49	252 (24.0%)	110 (26.8%)	63 (22%)	63 (20.9%)	16 (32.7%)
≥50	670 (63.9%)	274 (66.7%)	159 (55.4%)	224 (74.4%)	13 (26.5%)
Sex
Male	768 (73.3%)	291 (70.8%)	215 (74.9%)	219 (72.8%)	43 (87.8%)
Female	280 (26.7%)	120 (29.2%)	72 (25.1%)	82 (27.2%)	6 (12.2%)
Marital status
Single	220 (21.0%)	67 (16.3%)	84 (29.3%)	44 (14.6%)	25 (51%)
Married	537 (51.2%)	212 (51.6%)	139 (48.4%)	168 (55.8%)	18 (36.7%)
Divorced or widowed	291 (27.8%)	132 (32.1%)	64 (22.3%)	89 (29.6%)	6 (12.2%)
Ethnicity
Han	316 (30.2%)	114 (27.7%)	88 (30.7%)	96 (31.9%)	18 (36.7%)
Zhuang	691 (65.9%)	275 (66.9%)	190 (66.2%)	195 (64.8%)	31 (63.3%)
Others	41 (3.9%)	22 (5.4%)	9 (3.1%)	10 (3.3%)	0 (0%)
Education
Primary and below	553 (52.8%)	248 (60.3%)	131 (45.6%)	163 (54.2%)	11 (22.4%)
Junior high school	325 (31.0%)	129 (31.4%)	80 (27.9%)	101 (33.6%)	15 (30.6%)
Senior high school	89 (8.5%)	20 (4.9%)	33 (11.5%)	25 (8.3%)	11 (22.4%)
College and above	81 (7.7%)	14 (3.4%)	43 (15%)	12 (4%)	12 (24.5%)
Regional classification	
City	727 (69.4%)	316 (76.9%)	203 (70.7%)	181 (60.1%)	27 (55.1%)
Countryside	321 (30.6%)	95 (23.1%)	84 (29.3%)	120 (39.9%)	22 (44.9%)
Occupation
Farmer	802 (76.5%)	348 (84.7%)	198 (69%)	236 (78.4%)	20 (40.8%)
Others	246 (23.5%)	63 (15.3%)	89 (31%)	65 (21.6%)	29 (59.2%)
HBV-positive
No	947 (90.4%)	368 (89.5%)	266 (92.7%)	272 (90.4%)	41 (83.7%)
Yes	101 (9.6%)	43 (10.5%)	21 (7.3%)	29 (9.6%)	8 (16.3%)
HCV-positive
No	1034 (98.7%)	404 (98.3%)	283 (98.6%)	300 (99.7%)	47 (95.9%)
Yes	14 (1.3%)	7 (1.7%)	4 (1.4%)	1 (0.3%)	2 (4.1%)
STIs-positive
No	980 (93.5%)	382 (92.9%)	264 (92%)	291 (96.7%)	43 (87.8%)
Yes	68 (6.5%)	29 (7.1%)	23 (8%)	10 (3.3%)	6 (12.2%)
HIV transmission route	
Heterosexual transmission	970 (92.5%)	397 (96.6%)	243 (84.7%)	293 (97.3%)	37 (75.5%)
Homosexual transmission	70 (6.7%)	13 (3.2%)	41 (14.3%)	4 (1.3%)	12 (24.5%)
Others	8 (0.8%)	1 (0.2%)	3 (1%)	4 (1.3%)	0 (0%)
CD4^+^ T-cell count at baseline, cells/μL		
0–199	446 (42.6%)	203 (49.4%)	96 (33.4%)	128 (42.5%)	19 (38.8%)
≥200	602 (57.4%)	208 (50.6%)	191 (66.6%)	173 (57.5%)	30 (61.2%)
Year of diagnosis
2019	174 (16.6%)	82 (20%)	56 (19.5%)	31 (10.3%)	5 (10.2%)
2020	304 (29.0%)	137 (33.3%)	56 (19.5%)	96 (31.9%)	15 (30.6%)
2021	303 (28.9%)	111 (27%)	71 (24.7%)	100 (33.2%)	21 (42.9%)
2022	267 (25.5%)	81 (19.7%)	104 (36.2%)	74 (24.6%)	8 (16.3%)

^*a*^ Abbreviations: HBV, hepatitis B virus; HCV, hepatitis C virus; HIV, human immunodeficiency virus; STIs, sexually transmitted infections.

### Trends in HIV PDR prevalence and associated mutations

**Fig 2:**
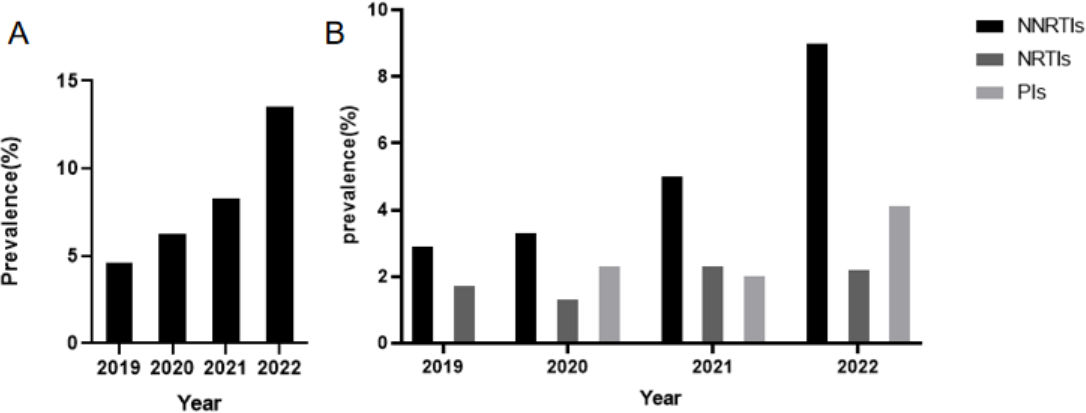
Trend and prevalence of PDR by year from 2019-2022 in Nanning City. (**A**) The trend of PDR prevalence from 2019 to 2022. (**B**) Prevalence of PDR to NNRTIs, NRTIs, and PIs from 2019 to 2022. PDR, Pretreatment Drug Resistance; NNRTIs, non-nucleoside reverse transcriptase inhibitors; NRTIs, nucleoside reverse transcriptase inhibitors; PIs, protease inhibitors.

The prevalence of PDR increased steadily from 4.6% in 2019 to 6.3% in 2020, 8.3% in 2021, and peaked at 13.5% in 2022, as shown in Fig. 2A. Regarding the types of drug resistance, NNRTI-associated PDR predominated throughout the study period, consistently exceeding resistance to NRTIs and PIs. Notably, the prevalence of NNRTI resistance reached its peak in 2022 (Fig. 2B).

**Fig 3:**
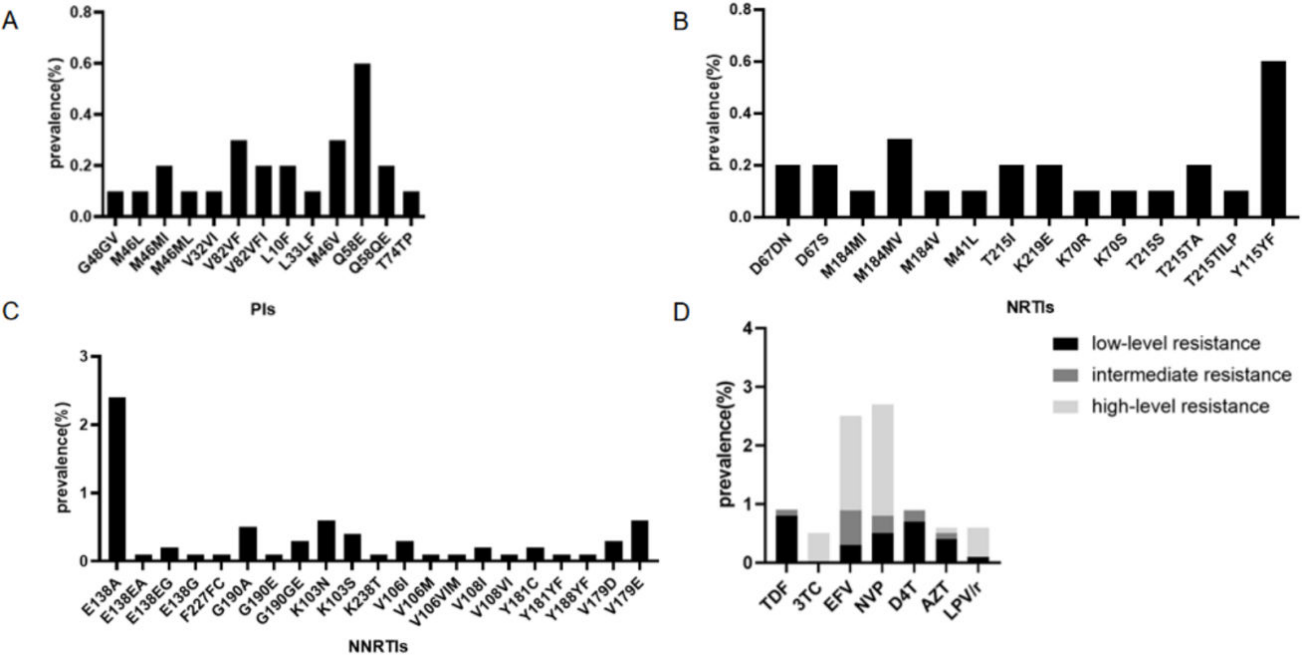
HIV PDR prevalence in Nanning City, 2019-2022. (**A–C**) DRM prevalence at different resistance levels. (**D**) HIV PDR prevalence by antiretroviral drug. PDR, pretreatment drug resistance; DRM, drug resistance mutation; NNRTIs, non-nucleoside reverse transcriptase inhibitors; NRTIs, nucleoside reverse transcriptase inhibitors; PIs, protease inhibitors; TDF, tenofovir disoproxil fumarate; 3TC, lamivudine; EFV, efavirenz; NVP, nevirapine; D4T, stavudine; AZT, zidovudine; LPV/r, lopinavir boosted with ritonavir.

Among PI-associated mutations, Q58E was the most prevalent (0.6%), followed by M46V (0.3%) and V82VF (0.3%) (Fig. 3A). For NRTI-associated mutations, Y115YF was the most prevalent (0.6%), followed by M184MV (0.3%) (Fig. 3B). The most NNRTI-associated mutation was E138A, with a prevalence of 2.4% (Fig. 3C).

Figure 3D illustrates the prevalence of resistance to these drugs, categorized into low, intermediate, and high levels of resistance. High-level resistance was predominantly observed for EFV (1.6%) and NVP (1.9%). From 2019 to 2022, the prevalence of PDR to EFV increased from 0.6% to 4.5%, and resistance to NVP rose from 1.1% to 4.9%.

Over the same period, NNRTI-associated PDR increased significantly (β = 2.00, *P* = 0.005). Although EFV (β = 1.14) and NVP (β = 1.11) exhibited upward trends, their slopes did not differ significantly from that of overall NNRTIs (*P* for interaction = 0.237 and 0.223) (Fig. S2).

### Characteristics of PDR TCs in molecular networks

**Fig 4:**
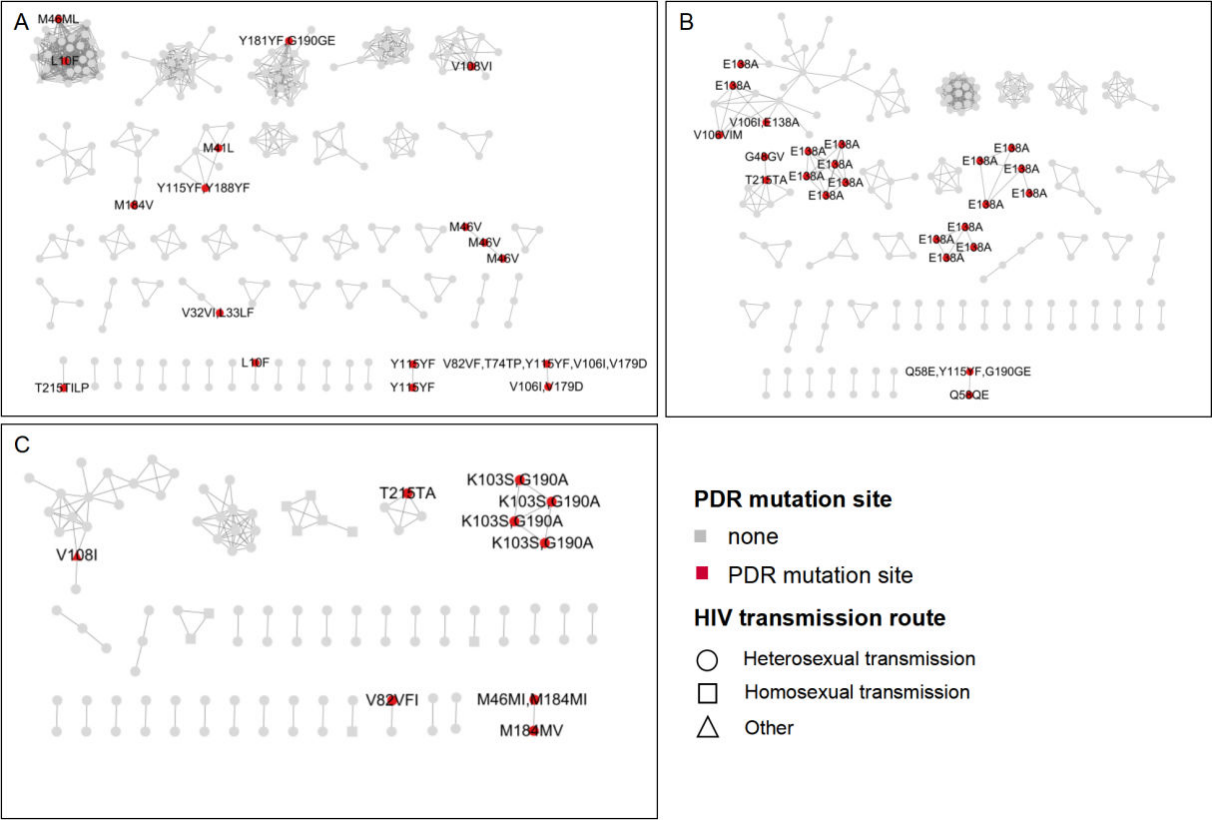
The molecular transmission networks of newly diagnosed individuals in Nanning City, 2019–2022. (**A**) The molecular network of CRF01_AE. (**B**) The molecular network of CRF08_BC. (**C**) The molecular network of CRF07_BC. Circles, squares, and triangles denote HIV transmission routes, and the frame colors indicate the PDR mutation sites. PDR, pretreatment drug resistance.

In the CRF01_AE network, three PDR TCs were observed. One cluster exhibited two Y115YF mutations, another displayed three M46V mutations, and the third contained two sequences harboring both V106I and V179D mutations. These clusters were primarily transmitted through heterosexual transmission (Fig. 4A).

In the CRF07_BC network, two PDR TCs were identified. One cluster included sequences with both K103S and G190A mutations, while another showed M184MV, M46MI, and M184MI mutations. These clusters were primarily transmitted through heterosexual contact (Fig. 4B).

In the CRF08_BC network, five PDR TCs were identified. The E138A mutation predominantly characterized four clusters, while one cluster showed Q58E and Q58QE mutations. Similar to the other subtypes, these clusters were primarily transmitted through heterosexual transmission (Fig. 4C).

### Factors associated with PDR

As shown in Table 2, multivariate logistic regression analysis revealed that the year of diagnosis was a significant factor associated with PDR. Individuals diagnosed in 2022 had a significantly higher risk of PDR compared to those diagnosed in 2019 (adjusted odds ratio [*aOR*] = 2.86, *95%* CI: 1.32–6.90). In addition, there was weak but statistically nonsignificant evidence suggesting that individuals infected with the CRF08_BC subtype were more likely to exhibit PDR (*aOR* = 1.64, *95%* CI: 0.96–2.84).

**TABLE 2:** Factors associated with PDR among newly diagnosed and ART-naïve individuals^*a*^

Characteristics	Univariable			Multivariable		
	cOR	95% CI	*P*	aOR	95% CI	*P*
Age (years)						
18–34	–^*b*^	–	–	–	–	–
35–49	1.05	(0.51–2.31)	0.899	–	–	–
≥50	0.92	(0.48–1.90)	0.801	–	–	–
Sex						
Male	–	–	–	–	–	–
Female	1.31	(0.81–2.08)	0.260	–	–	–
Marital status						
Single	–	–	–	–	–	–
Married	1.09	(0.62–2.00)	0.766	–	–	–
Divorced or widowed	1.17	(0.62–2.25)	0.627	–	–	–
Ethnicity						
Han	–	–	–	–	–	–
Zhuang	0.67	(0.43–1.07)	0.090	**0.61**	**(0.37–1.00**)	**0.046**
Others	0.43	(0.07–1.48)	0.253	0.47	(0.07–1.70)	0.322
Education						
Primary and below	–	–	–	–	–	–
Junior high school	0.82	(0.49–1.35)	0.445	–	–	–
Senior high school	1.02	(0.43–2.11)	0.969	–	–	–
College and above	0.97	(0.39–2.10)	0.948	–	–	–
Regional classification						
Countryside	–	–	–	–	–	–
City	1.73	(1.10–2.69)	0.016	1.58	(0.95–2.62)	0.074
Occupation						
Others	–	–	–	–	–	–
Farmer	1.05	(0.63–1.80)	0.863	1.65	(0.91–3.14)	0.111
HBV-positive						
No	–	–	–	–	–	–
Yes	0.67	(0.25–1.45)	0.352	–	–	–
HCV-positive						
No	–	–	–	–	–	–
Yes	0.84	(0.05–4.28)	0.865	–	–	–
STIs-positive						
No	–	–	–	–	–	–
Yes	0.49	(0.12–1.35)	0.230	0.44	(0.10–1.26)	0.182
HIV transmission route						
Heterosexual transmission	–	–	–	–	–	–
Homosexual transmission	1.04	(0.39–2.30)	0.924	–	–	–
Others	3.71	(0.54–16.40)	0.112	–	–	–
CD4^+^ T-cell count at baseline, cells/μL						
0–199	–	–	–	–	–	–
≥200	1.26	(0.81–1.99)	0.317	–	–	–
Year of diagnosis						
2019	–	–	–	–	–	–
2020	1.38	(0.61–3.42)	0.453	1.10	(0.47–2.77)	0.836
2021	1.87	(0.86–4.51)	0.135	1.50	(0.67–3.70)	0.346
2022	3.23	(1.54–7.65)	0.004	**2.86^*c*^**	**(1.32–6.90**)	**0.012**
Subtype						
CRF01_AE	–	–	–	–	–	–
CRF07_BC	1.01	(0.54–1.84)	0.979	0.88	(0.47–1.65)	0.701
CRF08_BC	1.93	(1.15–3.28)	0.014	1.64	(0.96–2.84)	0.072
Other subtypes	1.98	(0.71–4.79)	0.153	2.16	(0.74–5.51)	0.127

^*a*^ Abbreviations: aOR, adjusted odds ratio; cOR, crude odds ratio; HBV, hepatitis B virus; HCV, hepatitis C virus; HIV, human immunodeficiency virus; STIs, sexually transmitted infections.

^*b*^ –, no data available.

^*c*^ Bold indicates statistical significance (*P* < 0.05) in the multivariable analysis.

### Factors associated with transmission network clusters

Regarding molecular transmission network clustering, multivariate logistic regression analysis (Table 3) identified HIV subtype, age, ethnicity, and HIV transmission route as significant factors associated with the network. Individuals aged 50 years or older were more likely to be included in TCs (*aOR* = 1.75, *95%* CI: 1.05–2.93). In contrast, individuals with heterosexual transmission were more likely to cluster compared to those with homosexual transmission (*aOR* = 3.08, *95%* CI: 1.50–6.81). Moreover, infections with the CRF01_AE (*aOR* = 2.12, *95%* CI: 1.54–2.94) and CRF08_BC (*aOR* = 2.54, *95%* CI: 1.79–3.62) subtypes were significantly associated with higher clustering probability.

**TABLE 3:** Factors associated with clustering into the molecular transmission networks^*a*^

Characteristics	Univariable			Multivariable		
	cOR	95% CI	*P*	aOR	95% CI	*P*
Age (years)						
18–34	–^*b*^	–	–	–	–	–
35–49	1.49	(0.93–2.43)	0.103	0.81	(0.47–1.40)	0.457
≥50	3.18	(2.07–4.95)	0.000	**1.75**	**(1.05–2.93**)	**0.031**
Sex						
Male	–	–	–	–	–	–
Female	0.96	(0.73–1.27)	0.775	0.80	(0.60–1.08)	0.143
Marital status						
Single	–	–	–	–	–	–
Married	1.88	(1.35–2.63)	0.000	–	–	–
Divorced or widowed	1.75	(1.21–2.54)	0.003	–	–	–
Ethnicity						
Han	–	–	–	–	–	–
Zhuang	1.18	(0.89–1.55)	0.244	1.00	(0.74–1.35)	0.999
Others	1.90	(0.97–3.87)	0.065	**2.14^*c*^**	**(1.05–4.55**)	**0.041**
Education						
Primary and below	–	–	–	–	–	–
Junior high school	0.77	(0.58–1.02)	0.067	–	–	–
Senior high school	0.48	(0.30–0.78)	0.003	–	–	–
College and above	0.30	(0.17–0.51)	0.000	–	–	–
Regional classification						
Countryside	–	–	–	–	–	–
City	1.09	(0.83–1.43)	0.540	–	–	–
Occupation						
Others	–	–	–	–	–	–
Farmer	1.74	(1.29–2.37)	0.000	–	–	–
HBV-positive						
No	–	–	–	–	–	–
Yes	1.00	(0.66–1.55)	0.982	–	–	–
HCV-positive						
No	–	–	–	–	–	–
Yes	1.21	(0.38–4.12)	0.744	–	–	–
STIs-positive						
No	–	–	–	–	–	–
Yes	0.80	(0.48–1.34)	0.391	–	–	–
HIV transmission route						
Homosexual transmission	–	–	–	–	–	–
Heterosexual transmission	5.40	(2.87–11.09)	0.000	**3.08**	**(1.50–6.81**)	**0.003**
Others	4.27	(0.89–20.82)	0.063	2.63	(0.51–13.59)	0.236
CD4^+^ T-cell count at baseline, cells/μL						
0–199	–	–	–	–	–	–
≥200	0.95	(0.74–1.23)	0.710	–	–	–
Year of diagnosis						
2019	–	–	–	–	–	–
2020	0.93	(0.64–1.36)	0.718	–	–	–
2021	1.00	(0.68–1.46)	0.989	–	–	–
2022	0.88	(0.59–1.29)	0.505	–	–	–
PDR						
No	–	–	–	–	–	–
Yes	1.31	(0.83–2.09)	0.249	–	–	–
Subtype						
CRF07_BC	–	–	–	–	–	–
CRF01_AE	2.41	(1.77–3.29)	0.000	**2.12**	**(1.54–2.94**)	**0.000**
CRF08_BC	3.05	(2.19–4.28)	0.000	**2.54**	**(1.79–3.62**)	**0.000**

^*a*^ Abbreviations: aOR, adjusted odds ratio; cOR, crude odds ratio; HBV, hepatitis B virus; HCV, hepatitis C virus; HIV, human immunodeficiency virus; PDR, pretreatment drug resistance;STIs, sexually transmitted infections.

^*b*^ –, no data available.

^*c*^ Bold indicates statistical significance (*P* < 0.05) in the multivariable analysis.

### Factors associated with PDR TCs

As shown in Table 4, infection with the CRF_08BC subtype was significantly associated with inclusion in PDR TCs (*aOR* = 2.88, *95%* CI: 1.17–7.81). In addition, there was weak but statistically non-significant evidence that individuals diagnosed in 2022 were more likely to form PDR TCs (*aOR* = 4.49, *95%* CI: 1.17–29.58).

**TABLE 4:** Factors associated with clustering into the molecular transmission networks with PDR^*a*^

Characteristics	Univariable			Multivariable		
	cOR	95% CI	*P*	aOR	95% CI	*P*
Age (years)						
18–34	–^*b*^	–	–	–	–	–
35–49	0.44	(0.09–2.36)	0.305	–	–	–
≥50	0.71	(0.23–3.09)	0.590	–	–	–
Sex						
Male	–	–	–	–	–	–
Female	1.93	(0.91–3.98)	0.079	1.83	(0.83–3.92)	0.127
Marital status						
Single	–	–	–	–	–	–
Married	1.98	(0.66–8.53)	0.279	–	–	–
Divorced or widowed	1.39	(0.39–6.46)	0.636	–	–	–
Ethnicity						
Han	–	–	–	–	–	–
Zhuang	0.75	(0.35–1.65)	0.450	–	–	–
Others	0.49	(0.03–2.66)	0.498	–	–	–
Education						
Primary and below	–	–	–	–	–	–
Junior high school	1.21	(0.54–2.60)	0.624	–	–	–
Senior high school	1.12	(0.17–4.14)	0.884	–	–	–
College and above	0.84	(0.05–4.40)	0.867	–	–	–
Regional classification						
Countryside	–	–	–	–	–	–
City	0.38	(0.19–0.79)	0.009	0.57	(0.26–1.24)	0.151
Occupation						
Others	–	–	–	–	–	–
Farmer	0.90	(0.38–2.49)	0.829	–	–	–
HBV-positive						
No	–	–	–	–	–	–
Yes	0.30	(0.02–1.44)	0.240	–	–	–
STIs-positive						
No	–	–	–	–	–	–
Yes	0.53	(0.03–2.60)	0.537	–	–	–
CD4^+^ lymphocyte count at baseline, cells/μL						
0–199	–	–	–	–	–	–
≥200	1.49	(0.72–3.27)	0.297	–	–	–
Subtype						
CRF01_AE	–	–	–	–	–	–
CRF07_BC	2.01	(0.63–6.20)	0.220	1.40	(0.42–4.51)	0.569
CRF08_BC	3.64	(1.56–9.50)	0.004	**2.88^*c*^**	**(1.17–7.81**)	**0.027**
Year of diagnosis						
2019	–	–	–	–	–	–
2020	0.90	(0.15–6.96)	0.913	0.68	(0.11–5.37)	0.679
2021	3.14	(0.80–20.72)	0.146	2.28	(0.55–15.49)	0.309
2022	6.61	(1.83–42.38)	0.013	4.49	(1.17–29.58)	0.055

^*a*^ Abbreviations: aOR, adjusted odds ratio; cOR, crude odds ratio; HBV, hepatitis B virus; HCV, Hepatitis C Virus; HIV, human immunodeficiency virus; STIs, sexually transmitted infections.

^*b*^ –, no data available.

^*c*^ Bold indicates statistical significance (*P* < 0.05) in the multivariable analysis.

## DISCUSSION

This cross-sectional study provided comprehensive insights into PDR trends and molecular transmission networks in Nanning City, Guangxi Province. Our findings revealed an increasing trend of PDR from 2019 to 2022, with resistance against NNRTIs being the most prevalent and peaking in 2022. Notably, molecular transmission network analysis identified fully resistant clusters in which all sequences shared identical resistance mutations, indicating ongoing transmission of resistant strains. This phenomenon was particularly pronounced in the CRF08_BC subtype, which exhibited both higher rates of clustering and a greater potential for resistance transmission. These findings highlighted the rising burden of PDR and the urgent need for sustained surveillance and targeted intervention strategies. By addressing critical data gaps and advancing understanding of regional resistance dynamics, this study provided essential evidence to inform the design of targeted and effective treatment and prevention programs in Guangxi and other areas.

A previous national HIV molecular epidemiology survey reported a national average prevalence of 4.4% for drug resistance (3). In contrast, our study identified a higher prevalence of PDR, at 8.4%, among newly diagnosed individuals in Nanning, exceeding the previously reported 6.7% in 2017 (17). Although this prevalence remained moderate and below the WHO’s 10% threshold for public health concern (18), a steady increase in resistance was observed from 2019 to 2022. Notably, individuals newly diagnosed and ART-naïve in 2022 had a higher risk of PDR, suggesting a possible acceleration in the transmission of resistant HIV strains. Several factors may have contributed to this trend. First, the relaxation of COVID-19-related restrictions may have facilitated increased HIV transmission, including transmission of drug-resistant strains (19). Second, disruptions in HIV care during the pandemic, such as reduced access to routine viral load monitoring and delayed switching of failing ART regimens, may have contributed to the accumulation of resistance (20). Finally, the cumulative effect of transmitted resistance over the years may have reached a tipping point, resulting in a noticeable rise in 2022. In addition, local spread of drug resistance might have been driven by individuals experiencing ART failure or those with transmitted DRMs. Given that PDR could lead to virological failure (21), the accumulation of DRMs (22), and reduced efficacy of first-line regimens (23), ongoing surveillance of PDR in Guangxi is crucial. It is essential to improve the monitoring of resistance trends, promote adherence to ART, and use molecular tools to track the spread of resistant strains. These strategies are critical for mitigating drug resistance and ensuring the continued effectiveness of HIV treatment programs.

The highest prevalence of drug resistance in this study was observed to NNRTIs, particularly EFV and NVP, consistent with previous studies (24, 25). NNRTIs are known for their significant treatment efficacy (26), low pill burden, and favorable tolerability, which collectively improve adherence to ART. However, their widespread use has facilitated the emergence and persistence of drug-resistant mutations, which are easily detected due to their long-term stability in the viral population. HIV-1 is characterized by a low genetic barrier to NNRTI resistance, whereby a single mutation at the binding site can lead to drug resistance (27). A previous study has indicated that mutations in the HIV reverse transcriptase (RT) gene, beyond the currently defined resistance-associated mutations, may also contribute to NNRTI resistance *in vivo* (28). Long-standing HIV epidemics may further compound the accumulation of resistant mutations over time. According to WHO guidelines, when the prevalence of NNRTI-related PDR reaches or exceeds 10%, switching to a non-NNRTI-based first-line regimen is advised. These findings underscore the importance of adaptive ART strategies as resistance thresholds are approached, thereby ensuring sustained treatment efficacy and preventing the transmission of further resistance.

Consistent with previous studies in Guangxi (2), Sichuan (29), and Anhui (30) provinces, our study found that individuals aged 50 years or older were more likely to be part of molecular TCs. This pattern may reflect the relatively stable geographic locations and limited mobility of older adults, which restricts the broader dissemination of HIV-1 within this subgroup. Furthermore, older individuals often have a lower awareness of HIV-related risks (31) and are more likely to engage in commercial sex, exacerbating local transmission. Geographic hotspots arising from commercial sexual behaviors between older men and female sex workers play a significant role in driving the local HIV-1 epidemic (32). Given these observations, urgent efforts are required to curb ongoing transmission among older adults. Specifically, public health responses should prioritize the identification of TCs and the implementation of localized, targeted interventions aimed at disrupting transmission pathways in this high-risk and often underserved population.

We identified three PDR clusters in CRF01_AE, two in CRF07_BC, and five in CRF08_BC. These findings suggested that individuals within the same cluster are closely linked in terms of transmission dynamics. In this study, CRF08_BC exhibited a higher likelihood of PDR clustering compared to CRF01_AE. CRF08_BC has become a predominant strain among heterosexuals and injection drug users in Southern China. Notably, the transmission route for five clusters within the CRF08_BC subtype was primarily heterosexual contact. A previous study found that heterosexual transmission has surpassed injection drug use as the leading mode of CRF08_BC infection nationwide (33). We also observed a high prevalence of the E138A mutation, particularly within CRF08_BC strains, which aligns with findings from an earlier study in China (34), suggesting that E138A may be a signature mutation of this subtype. The identification of PDR clusters in this study underscores the importance of ongoing molecular network surveillance among newly diagnosed individuals, enabling precise and targeted interventions aimed at containing PDR dissemination.

This study had several limitations. First, the cross-sectional design limited our ability to assess the temporal dynamics of HIV transmission. Second, molecular network analysis does not establish direct transmission links between genetically connected individuals, and self-reported data, such as modes of sexual contact, are subject to information bias. Third, although all participants were newly diagnosed and ART-naïve, we were unable to verify whether any had previously used antiretroviral drugs for non-HIV indications. The limitation could have resulted in an incomplete representation of the transmission network and the exclusion of relevant individuals. Future studies involving larger and more diverse populations, with available contact and behavioral information, are needed to design effective interventions for high-risk groups. Regardless, given the significant risk of PDR, it is essential to integrate these findings into both treatment and prevention strategies.

### Conclusion

This study revealed that the prevalence of PDR in Nanning was moderate between 2019 and 2022. The most common drug-resistant mutations were associated with resistance to NNRTIs, especially involving EFV and NVP. The E138A mutation was frequently detected in the CRF08_BC subtype, which showed increased PDR clustering within transmission networks. These findings underscore the utility of molecular network analysis for monitoring PDR and identifying targeted opportunities for intervention. Continued surveillance, particularly of NNRTI resistance and key mutations such as E138A, along with strengthened control measures, is essential to limit the emergence and transmission of PDR. These will be critical for guiding the selection and optimization of ART regimens in Guangxi.

## MATERIALS AND METHODS

### Study setting and population

A cross-sectional survey using a convenience sampling method was conducted across the nine counties and districts of Nanning City, Guangxi Province, from 1 January 2019 to 31 December 2022. During each survey period, staff at the recruitment sites recruited individuals newly diagnosed with HIV. To construct the molecular transmission network, we recruited more than 60% newly diagnosed HIV patients each year.

The participant inclusion criteria were as follows: (i) aged 18 years or older; (ii) newly diagnosed with HIV and ART naive; (iii) residence in Nanning for more than three months before diagnosis; and (iv) ability to provide verbal and written informed consent in Mandarin.

Venous blood samples (10 mL) were collected from each participant. Plasma was separated by centrifugation and stored in aliquots at −80°C for further analysis. Demographic and epidemiological information, including sex, ethnicity, age, education, occupation, marital status, year of diagnosis, and transmission route, was obtained through a structured questionnaire administered by trained personnel.

### Laboratory testing

HIV-1 RNA was extracted from plasma samples using the High Pure Viral RNA Kit (Roche, Germany). An in-house RT nested PCR was used to amplify a 1,061 bp fragment of the pol gene, covering the full-length protease (PR, 99 codons) and the first 299 codons of the RT gene. Reverse transcription and the first-round PCR were performed using a Prime Script One Step RT-PCR Kit (Takara, Dalian, China) under the following thermal cycling conditions: 50°C for 30 minutes, 94°C for 5 minutes, followed by 30 cycles of 94°C for 30 seconds, 55°C for 30 seconds, and 72°C for 2 minutes, with a final extension at 72°C for 10 minutes.

For the first-round PCR, two forward primers were used: F1a (5′-TGAARGAITGYACTGARAGRCAGGCTAAT-3′, HXB2 positions 2,057–2,085) and F1b (5′-ACTGARAGRCAGGCTAATTTTTTAG-3′, HXB2 positions 2,068–2,092), along with the reverse primer RT-R1 (5′-ATCCCTGCATAAATCTGACTTGC-3′, HXB2 positions 3,370–3,348).

Nested PCR was performed in a 50 µL reaction volume under the following conditions: 94°C for 5 minutes; 30 cycles of 94°C for 30 seconds, 63°C for 30 seconds, and 72°C for 2.5 minutes, and a final extension at 72°C for 10 minutes. The nested PCR used the forward primer PRT-F2 (5′-CTTTARCTTCCCTCARATCACTCT-3′, corresponding to HXB2 positions 2,243–2,266) and the reverse primer RT-R2 (5′-CTTCTGTATGTCATTGACAGTCC-3′, corresponding to HXB2 positions 3,326-3,304).

PCR products were confirmed by agarose gel electrophoresis, and positive amplicons were sequenced by Sangon Biotech (Shanghai, China) using the Applied Biosystems 3730XL Genetic Analyzer. Raw chromatogram data were assembled and cleaned using Sequencher 5.4.6.

### Sequence processing and quality control

Quality control was performed using the quality control tool from the Los Alamos National Laboratory HIV Sequence Database (https://www.hiv.lanl.gov) to identify and exclude sequences with mixed bases >5% or premature stop codons. All nucleotide sequences were aligned using the HIV Align tool (https://www.hiv.lanl.gov) and manually edited in BioEdit (version 7.0.9.0).

### Genotypic resistance analysis

DRMs and PDR were performed using the Genotypic Resistance Interpretation tool provided by the Stanford University HIV Drug Resistance Database (version 8.9; https://hivdb.stanford.edu). DRMs were classified based on their ability to confer resistance to nucleoside RT inhibitors (NRTIs), NNRTIs, and protease inhibitors (PIs). PDR was defined based on resistance to one or more of the following antiretroviral drugs: seven NRTIs (abacavir [ABC], zidovudine [AZT], emtricitabine [FTC], lamivudine [3TC], tenofovir [TDF], stavudine [D4T], and didanosine [DDI]), five NNRTIs (doravirine [DOR], EFV, etravirine [ETR], NVP, and rilpivirine [RPV]), and eight PIs (atazanavir/r [ATV/r], darunavir/r [DRV/r], lopinavir/r [LPV/r], fosamprenavir/r [FPV/r], indinavir/r [IDV/r], nelfinavir [NFV], saquinavir/r [SQV/r], and tipranavir/r [TPV/r]). Based on the genotypic susceptibility scores, PDR was classified as high-level (score >60), intermediate-level (30–59), or low-level (15–29).

### Molecular transmission network inference

Genetic distances were calculated for three predominant HIV-1 subtypes (CRF01_AE, CRF07_BC, and CRF08_BC) using the HIV TRACE tool (https://github.com/veg/hivtrace). To achieve a high-resolution molecular network, genetic distance thresholds for all sequences and the three major subtypes were optimized to maximize the number of molecular clusters, prevent the formation of oversized clusters, and more accurately identify potential transmission relationships (35). The optimal genetic distance threshold was defined as the value that yielded the most significant number of TCs. In this study, the optimal genetic distance thresholds were 0.015 for CRF01_AE, 0.008 for CRF07_BC, and 0.013 for CRF08_BC, ensuring both resolution and interpretability of the transmission networks (Fig. S1). A PDR cluster was defined as a network containing two or more identical DRMs. The HIV-1 genetic transmission network was visualized and analyzed using Cytoscape version 3.10.0.

### Statistical analysis

Statistical analysis was performed using R version 4.3.1. Quantitative data were presented as mean ± standard deviation for normally distributed variables and as median (interquartile range) for non-normally distributed variables. Frequencies and percentages were used to describe categorical variables. Univariate and multivariate logistic regression analyses were used to identify factors associated with PDR, transmission network clustering, and clustering within PDR transmission networks. A backward stepwise selection method was employed to determine the final model. *P*-values <0.05 were considered statistically significant.

To examine whether the temporal trends of PDR differed among NNRTIs overall and the specific drugs EFV and NVP, we fitted a linear regression model that included an interaction term between year and drug group. A statistically significant interaction term indicates that the temporal trend (i.e., slope of change) differs among drug groups. This approach allows for direct assessment of whether the rate of change in PDR differs between specific drug subgroups. The observed and fitted trends were visualized using the ggplot2 package.

## Data Availability

HIV-1 sequence data generated in this study have been deposited in GenBank and are available under accession numbers PX133190–PX134237.

## SUPPLEMENTAL MATERIAL

Supplemental material spectrum.03149-24-s0001.pdf Fig. S1 and S2; Table S1.

ASM does not own the copyrights to Supplemental Material that may be linked to, or accessed through, an article. The authors have granted ASM a non-exclusive, world-wide license to publish the Supplemental Material files. Please contact the corresponding author directly for reuse.
