# Supplementary material for: Molecular epidemiology and pretreatment drug resistance of HIV-1 among newly diagnosed individuals in Nanning City, Guangxi, China
Source: Microbiol Spectr. 2025 Sep 12;13(10):e03149-24. doi: 10.1128/spectrum.03149-24 (PMC12502766; doi:10.1128/spectrum.03149-24)
Supplement: Supplemental material — Fig. S1 and S2; Table S1. [file spectrum.03149-24-s0001.pdf]

Supplemental Material

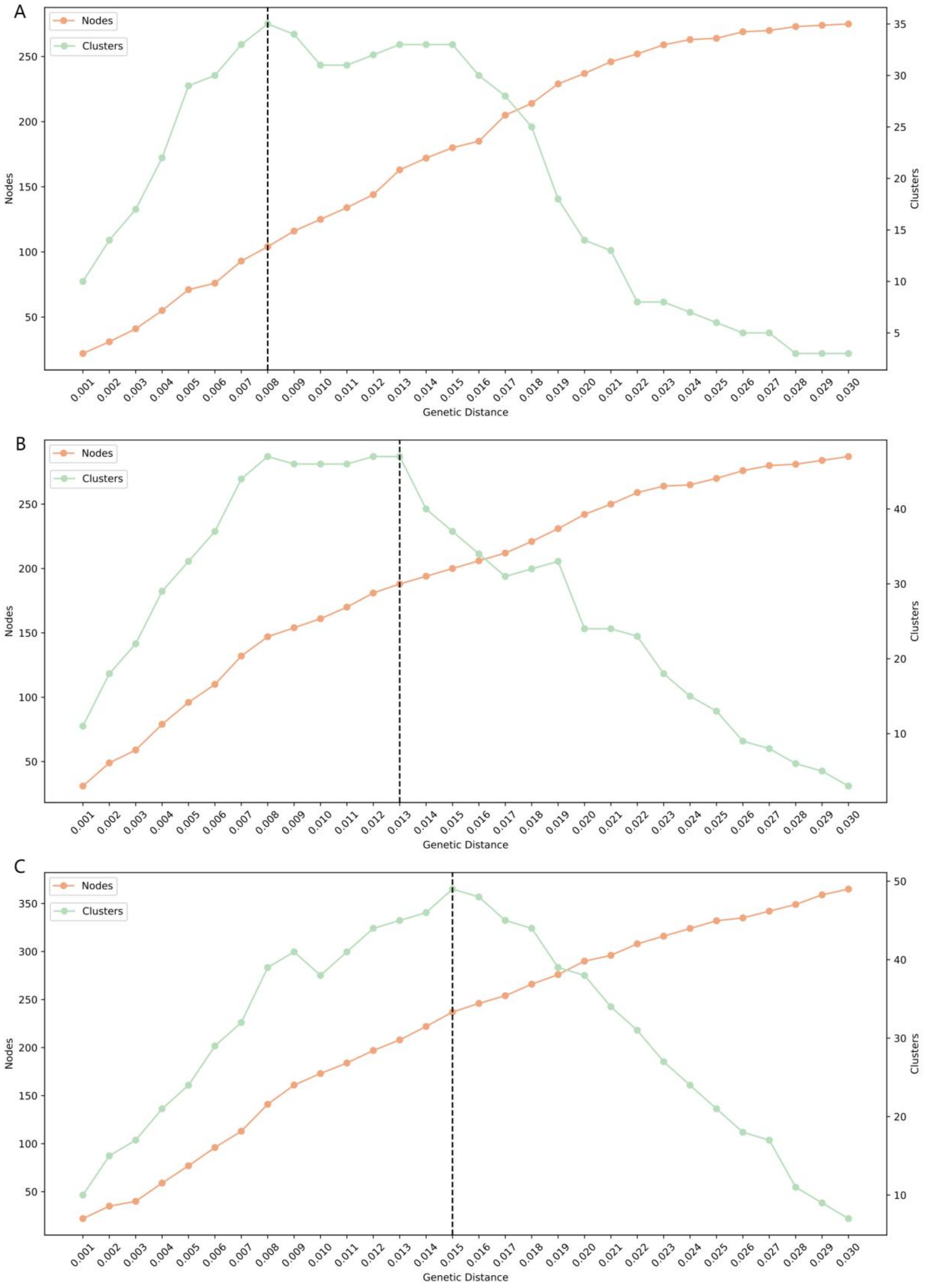

Supplementary Figure 1. Determination of the optimal genetic distance threshold for molecular clustering. (A) CRF07_BC; (B) CRF08_BC; (C) CRF01_AE.

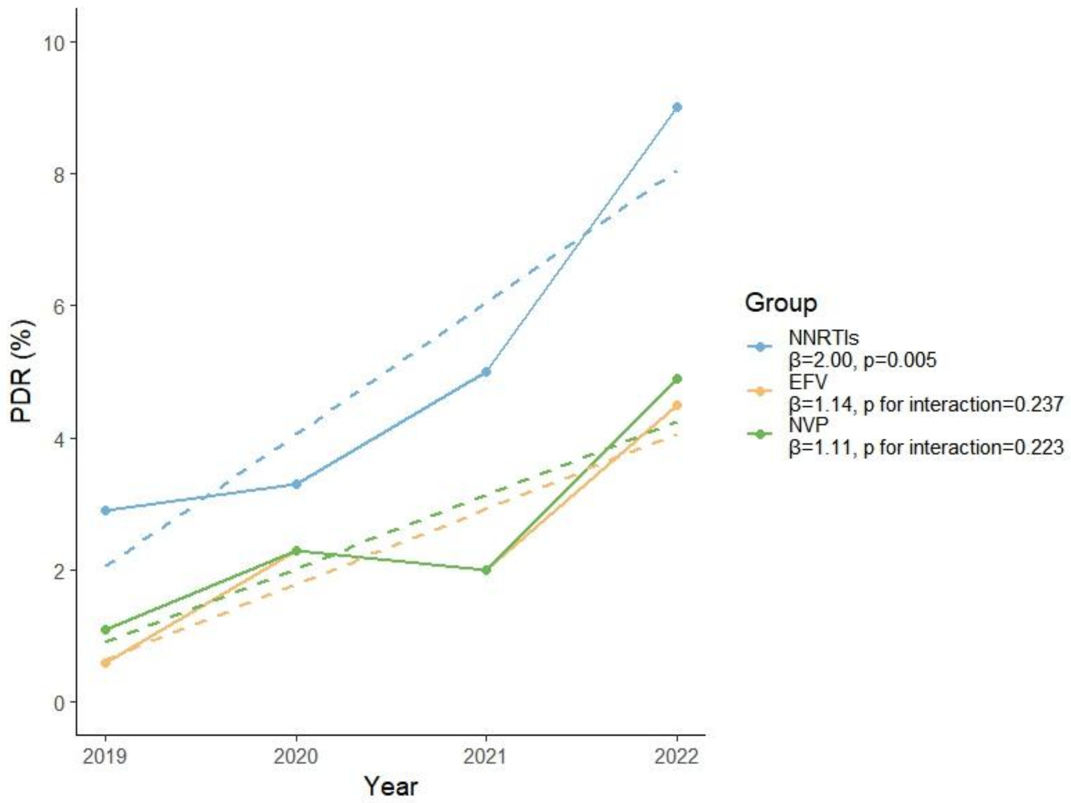

Supplementary Figure 2. Trends in PDR to NNRTIs, EFV, and NVP from 2019 to 2022. Solid lines represent observed annual PDR rates; dashed lines indicate fitted linear regression trends. PDR, Pretreatment Drug Resistance; NNRTIs, Non-Nucleoside Reverse Transcriptase Inhibitors; EFV, efavirenz; NVP, nevirapine.

Supplementary Table 1. Factors Influencing the Inclusion of Individuals into the E138A Molecular Transmission Clusters.

Characteristics		Univariable			Multivariable	
	cOR	*95% CI*	*P*	*aOR*	*95% CI*	*P*
Age (years)						
18-34						
35-49	1.11	(0.14-22.68)	0.932			
≥50	1.24	(0.24-22.82)	0.837			
Sex						
Male						
Female	1.92	(0.69-5.10)	0.192			
Marital status						
Single						
Married	3.16	(0.60-58.17)	0.273			
Divorced or widowed	2.69	(0.43-51.86)	0.370			
Ethnicity						
Han						
Zhuan	1.20	(0.41-4.34)	0.755			
Others	1.40	(0.07-9.86)	0.769			
Regional classification						
Countryside						
City	1.31	(0.46-4.69)	0.643			
Occupation						
Others						
Farmer	1.70	(0.47-10.87)	0.486			
$CD4^+$ T cell count at baseline, cells/μL						
0-199						
≥200	1.79	(0.66-5.70)	0.278			
Year of diagnosis						
2019						
2020	0.35	(0.02-3.68)	0.392	0.35	(0.02-3.68)	0.392
2021	1.77	(0.38-12.54)	0.497	1.77	(0.38-12.54)	0.497
2022	3.36	(0.85-22.32)	0.126	3.36	(0.85-22.32)	0.126

Abbreviations: cOR, crude Odds Ratio; aOR, adjusted Odds Ratio
